# Supplementary material for: Investigating the Crime Scene—Molecular Signatures in Inflammatory Bowel Disease
Source: Int J Mol Sci. 2023 Jul 7;24(13):11217. doi: 10.3390/ijms241311217 (PMC10342864; doi:10.3390/ijms241311217)
Supplement: Supplementary file 1 [file ijms-24-11217-s001.zip › ijms-2453742-supplementary.pdf]

Table S1. Examples of how gut bacteria exert a pro- or anti-inflammatory impact

Bacterial species	Known mechanisms
BACTERIA WITH AN ALLEVIATING IMPACT	
Firmicutes	
Faecalibacterium prausnitzii[1-3]	Butyrate production, induction of regulatory T cells, energy supply to intestinal epithelium
Roseburia spp. *[1, 4]*	Butyrate production
Clostridium clusters IV and XIVa[5]	Butyrate production
Dialister invisus. [2, 5]	Acetate/proprionate production
Ruminococcus spp. *[6-9]*	Butyrate production, Mucus degradation
Bacteroidetes	
Bacteroides fragilis[10, 11]	Induction of IL-10 production from regulatory T cells, suppression of IL-17 production
Actinobacteria	
Bifidobacterium spp.*[5, 12]*	Acetate/proprionate production, induction of IL-10 production from regulatory T cells
Verrucomicrobia	
Akkermansia muciniphila[7]	Acetate/proprionate production, mucus changes
Proteobacteria	
Helicobacter pylori[13]	Induction of tolerogenic dendritic and regulatory T cells
BACTERIA WITH AN ENHANCING IMPACT	
Bacteroidetes	
Prevotella spp.*[14]*	Hampers acetate production
Bacteroides spp. *[15]*	Cleavage of junctional protein, induction of epithelial-derived IL-8, epithelial invasion
Proteobacteria	
Escherichia coli[1, 16]	Adhesion and invasion of colon epithelium, LPS release at cell death
Shigella spp.*[1, 16]*	Adhesion and invasion of colon epithelium, LPS release at cell death
Fusobacteria	
Fusobacterium spp. *[17]*	Adhesion and invasion of colon epithelium, induction of epithelial-derived IL-8 and TNF-α

1. Schirmer, M.; Garner, A.; Vlamakis, H.; Xavier, R. J., Microbial genes and pathways in inflammatory bowel disease. *Nature reviews. Gastroenterology & hepatology* **2019,** 17, (8), 497-511.

2. Joossens, M.; Huys, G.; Cnockaert, M.; De Preter, V.; Verbeke, K.; Rutgeerts, P.; Vandamme, P.; Vermeire, S., Dysbiosis of the faecal microbiota in patients with Crohn's disease and their unaffected relatives. *Gut* **2011,** 60, (5), 631.
3. Sedwick, C., A Novel Regulatory T Cell Population in the Gut. *PLOS Biology* **2014,** 12, (4), e1001834.
4. Machiels, K.; Joossens, M.; Sabino, J.; De Preter, V.; Arijs, I.; Eeckhaut, V.; Ballet, V.; Claes, K.; Van Immerseel, F.; Verbeke, K.; Ferrante, M.; Verhaegen, J.; Rutgeerts, P.; Vermeire, S., A decrease of the butyrate-producing species Roseburia hominis and Faecalibacterium prausnitzii defines dysbiosis in patients with ulcerative colitis. *Gut* **2014,** 63, (8), 1275-83.
5. Joossens, M.; Huys, G.; Cnockaert, M.; De Preter, V.; Verbeke, K.; Rutgeerts, P.; Vandamme, P.; Vermeire, S., Dysbiosis of the faecal microbiota in patients with Crohn's disease and their unaffected relatives. *Gut* **2011,** 60, (5), 631-7.
6. Yu, S.; Balasubramanian, I.; Laubitz, D.; Tong, K.; Bandyopadhyay, S.; Lin, X.; Flores, J.; Singh, R.; Liu, Y.; Macazana, C.; Zhao, Y.; Béguet-Crespel, F.; Patil, K.; Midura-Kiela, M. T.; Wang, D.; Yap, G. S.; Ferraris, R. P.; Wei, Z.; Bonder, E. M.; Häggblom, M. M.; Zhang, L.; Douard, V.; Verzi, M. P.; Cadwell, K.; Kiela, P. R.; Gao, N., Paneth Cell-Derived Lysozyme Defines the Composition of Mucolytic Microbiota and the Inflammatory Tone of the Intestine. *Immunity* **2020,** 53, (2), 398-416.e8.
7. Png, C. W.; Linden, S. K.; Gilshenan, K. S.; Zoetendal, E. G.; McSweeney, C. S.; Sly, L. I.; McGuckin, M. A.; Florin, T. H., Mucolytic bacteria with increased prevalence in IBD mucosa augment in vitro utilization of mucin by other bacteria. *Am.J.Gastroenterol.* **2010,** 105, (11), 2420-2428.
8. Morrison, D. J.; Preston, T., Formation of short chain fatty acids by the gut microbiota and their impact on human metabolism. *Gut microbes* **2016,** 7, (3), 189-200.
9. Crost, E. H.; Tailford, L. E.; Monestier, M.; Swarbreck, D.; Henrissat, B.; Crossman, L. C.; Juge, N., The mucin-degradation strategy of Ruminococcus gnavus: The importance of intramolecular trans-sialidases. *Gut microbes* **2016,** 7, (4), 302-312.
10. Round, J. L.; Mazmanian, S. K., Inducible Foxp3+ regulatory T-cell development by a commensal bacterium of the intestinal microbiota. *Proceedings of the National Academy of Sciences of the United States of America* **2010,** 107, (27), 12204-12209.
11. Mazmanian, S. K.; Round, J. L.; Kasper, D. L., A microbial symbiosis factor prevents intestinal inflammatory disease. *Nature* **2008,** 453, (7195), 620-5.
12. Jeon, S. G.; Kayama, H.; Ueda, Y.; Takahashi, T.; Asahara, T.; Tsuji, H.; Tsuji, N. M.; Kiyono, H.; Ma, J. S.; Kusu, T.; Okumura, R.; Hara, H.; Yoshida, H.; Yamamoto, M.; Nomoto, K.; Takeda, K., Probiotic Bifidobacterium breve induces IL-10-producing Tr1 cells in the colon. *PLoS pathogens* **2012,** 8, (5), e1002714.
13. Yu, Y.; Zhu, S.; Li, P.; Min, L.; Zhang, S., Helicobacter pylori infection and inflammatory bowel disease: a crosstalk between upper and lower digestive tract. *Cell Death Dis* **2018,** 9, (10), 961-961.
14. Iljazovic, A.; Roy, U.; Gálvez, E. J. C.; Lesker, T. R.; Zhao, B.; Gronow, A.; Amend, L.; Will, S. E.; Hofmann, J. D.; Pils, M. C.; Schmidt-Hohagen, K.; Neumann-Schaal, M.; Strowig, T., Perturbation of the gut microbiome by Prevotella spp. enhances host susceptibility to mucosal inflammation. *Mucosal Immunology* **2021,** 14, (1), 113-124.
15. Yu, L. C.-H., Microbiota dysbiosis and barrier dysfunction in inflammatory bowel disease and colorectal cancers: exploring a common ground hypothesis. *Journal of Biomedical Science* **2018,** 25, (1), 79.
16. Subramanian, S.; Campbell, B. J.; Rhodes, J. M., Bacteria in the pathogenesis of inflammatory bowel disease. *Current opinion in infectious diseases* **2006,** 19, (5), 475-484.
17. Strauss, J.; Kaplan, G. G.; Beck, P. L.; Rioux, K.; Panaccione, R.; Devinney, R.; Lynch, T.; Allen-Vercoe, E., Invasive potential of gut mucosa-derived Fusobacterium nucleatum positively correlates with IBD status of the host. *Inflamm Bowel Dis* **2011,** 17, (9), 1971-8.

Table S2. Inflammatory bowel disease–associated genes involved in gut barrier immunology and microbiome handling

Gene Symbol	Genomic location*	Gene name	CD/UC/ IBD/ VEO-IBD	Genes in GWAS region	Intestinal epithelial cell expression (proteinatlas) (first cell type=most expressed)	Known function in epithelial cells	Coding variant**	REFs
ALPI	chr2:232,456,125-232,460,753	Alkaline phosphatase, intestinal	VEO-IBD		Proximal enterocytes (enriched), distal enterocytes, paneth cells, intestinal goblet cells	Regulates the response to LPS[1]. Regulates tight junction, lyzozyme secretion, autophagy and mucus secretion[2].	ALPI(A360V), ALPI(Q439X)	[3]
ATG16L1	chr2:233,210,051-233,295,674	Autophagy related 16 like 1	CD	INPP5D, ATG16L1,SAG	Distal enterocytes, proximal enterocytes, paneth cells, intestinal goblet cells	Regulates the Paneth cell granule exocytosis pathway[4]. Regulates Paneth cell endoplasmic reticulum stress[5]. Regulates autophagy[6].	ATG16L1(T300A)	[7], [8]
IFIH1	chr2:162,267,074-162,318,684	Interferon induced with helicase C domain 1	UC	FAP,IFIH1	Proximal enterocytes, intestinal goblet cells, distal enterocytes, paneth cells	Implicated in the response to viruses[9].	IFIH1(I923V)	[10], [8]
NOD2	chr16:50,693,588-50,734,041	Nucleotide binding oligomerization domain containing 2	CD	CYLD,NOD2,SNX20	Expressed in Caco-2 cells	Regulates anti-microbial peptide secretion in Paneth cells[11]. Regulates autophagy[12]. Regulates crypt survival[13]. Regulates ROS production[14]. Regulates mucus production by goblet cells[15]. Regulates the recognition of intestinal microflora[15]. Regulates apoptosis[16]. Regulates cell proliferation[15]. Regulates the response to endoplasmic reticulum stress[17].	NOD2(R702W), NOD2(V793M), NOD2(S431L), NOD2(N289S), NOD2(N852S), NOD2(N872S), NOD2(fs1007insC), NOD2(G908R)	[18], [19], [8]
ADAM17	chr2:9,488,486-9,556,732	ADAM metallopeptidase domain 17	VEO-IBD		Distal enterocytes, paneth cells, intestinal goblet cells, proximal enterocytes	Modulates the response to TNF-α and EGFR[20]. Regulates tight junctions and cell shedding[21]. Regulates apoptosis in mouse[22]. Regulates mucus production in mouse[23]. Regulates cell proliferation[23].	ADAM17 (p.Val673Ile)	[24], [25]
TTC7A	chr2:46,916,157-47,076,137	Tetratricopeptide repeat protein 7A	VEO-IBD		Proximal enterocytes, distal enterocytes, paneth cells, intestinal goblet cells	Binds to PI4KIIIalpha and recruits it to the plasma membrane to produce PI4P[26],[27]. PI4P is implicated in cell polarization and cell survival[28].	E71K, A832T, and Q526X	[27]

C1orf106	chr1:200,891,048-200,915,742	Innate immunity activator	IBD	CAMSAP2,KIF21B,GPR25,CACNA1S,C1orf106	Expressed in Caco-2 cells	Regulates epithelial permeability through the regulation of adherens junction stability[29]. Regulates IL-1B signal transduction[30].	C1orf106(p.Y333F)	[31], [29]
FUT2	chr19:48,695,971-48,705,951	Fucosyltransferase 2	CD	NTN5,RASIP1,FUT2,MAMSTR,FUT1,IZUMO1	Proximal enterocytes (enriched), distal enterocytes (enriched), paneth cells (enriched), intestinal goblet cells	Intestinal epithelial cell fucosylation[32].	FUT2(W154X), FUT2(G258S)	[33-36], [8]
RNF186	chr1:19,814,029-19,815,283	Ring finger protein 186	UC	OTUD3, RNF186, TMCO4	Paneth cells (enriched), intestinal goblet cells (enriched), distal enterocytes (enriched), proximal enterocytes	E3 ubiquitin-protein ligase that regulates autophagy[37]. Regulates cell proliferation and migration[38]. Regulates endoplasmic reticulum stress[39].	RNF186(R179X); RNF186(p.Ala64Thr)	[39, 40], [41]
GUCY2C	chr12:14,612,632-14,696,599	Guanylate cyclase 2C	VEO-IBD		Paneth cells (enriched), proximal enterocytes (enriched), intestinal goblet cells (enriched), distal enterocytes (enriched)	Regulates water absorption[42]. Regulates cell proliferation, stem cell differentiation, DNA repair, and endoplasmic reticulum stress[42]. Regulates apoptosis[43].	GUCY2C(G549S), GUCY2C (F525L)	[44], [3]

*hg38, **both aminoacid and gene variant coding is used
abbreviations: LPS, lipopolysaccharide

Reference List

1. Parlato, M.; Charbit-Henrion, F.; Pan, J.; Romano, C.; Duclaux-Loras, R.; Le Du, M. H.; Warner, N.; Francalanci, P.; Bruneau, J.; Bras, M.; Zarhrate, M.; Bègue, B.; Guegan, N.; Rakotobe, S.; Kapel, N.; De Angelis, P.; Griffiths, A. M.; Fiedler, K.; Crowley, E.; Ruemmele, F.; Muise, A. M.; Cerf-Bensussan, N., Human ALPI deficiency causes inflammatory bowel disease and highlights a key mechanism of gut homeostasis. *EMBO Mol Med* **2018,** 10, (4).
2. Singh, S. B.; Lin, H. C., Role of Intestinal Alkaline Phosphatase in Innate Immunity. *Biomolecules* **2021,** 11, (12), 1784-1795.
3. Crowley, E.; Warner, N.; Pan, J.; Khalouei, S.; Elkadri, A.; Fiedler, K.; Foong, J.; Turinsky, A. L.; Bronte-Tinkew, D.; Zhang, S.; Hu, J.; Tian, D.; Li, D.; Horowitz, J.; Siddiqui, I.; Upton, J.; Roifman, C. M.; Church, P. C.; Wall, D. A.; Ramani, A. K.; Kotlarz, D.; Klein, C.; Uhlig, H.; Snapper, S. B.; Gonzaga-Jauregui, C.; Paterson, A. D.; McGovern, D. P. B.; Brudno, M.; Walters, T. D.; Griffiths, A. M.; Muise, A. M., Prevalence and Clinical Features of Inflammatory Bowel Diseases Associated With Monogenic Variants, Identified by Whole-Exome Sequencing in 1000 Children at a Single Center. *Gastroenterology* **2020,** 158, (8), 2208-2220.
4. Cadwell, K.; Liu, J. Y.; Brown, S. L.; Miyoshi, H.; Loh, J.; Lennerz, J. K.; Kishi, C.; Kc, W.; Carrero, J. A.; Hunt, S.; Stone, C. D.; Brunt, E. M.; Xavier, R. J.; Sleckman, B. P.; Li, E.; Mizushima, N.; Stappenbeck, T. S.; Virgin, H. W. t., A key role for autophagy and the autophagy gene Atg16l1 in mouse and human intestinal Paneth cells. *Nature* **2008,** 456, (7219), 259-63.

5. Deuring, J. J.; Fuhler, G. M.; Konstantinov, S. R.; Peppelenbosch, M. P.; Kuipers, E. J.; de Haar, C.; van der Woude, C. J., Genomic ATG16L1 risk allele-restricted Paneth cell ER stress in quiescent Crohn's disease. *Gut* **2014,** 63, (7), 1081-91.
6. Salem, M.; Ammitzboell, M.; Nys, K.; Seidelin, J. B.; Nielsen, O. H., ATG16L1: A multifunctional susceptibility factor in Crohn disease. *Autophagy* **2015,** 11, (4), 585-94.
7. Rioux, J. D.; Xavier, R. J.; Taylor, K. D.; Silverberg, M. S.; Goyette, P.; Huett, A.; Green, T.; Kuballa, P.; Barmada, M. M.; Datta, L. W.; Shugart, Y. Y.; Griffiths, A. M.; Targan, S. R.; Ippoliti, A. F.; Bernard, E. J.; Mei, L.; Nicolae, D. L.; Regueiro, M.; Schumm, L. P.; Steinhart, A. H.; Rotter, J. I.; Duerr, R. H.; Cho, J. H.; Daly, M. J.; Brant, S. R., Genome-wide association study identifies new susceptibility loci for Crohn disease and implicates autophagy in disease pathogenesis. *Nat Genet* **2007,** 39, (5), 596-604.
8. Huang, H.; Fang, M.; Jostins, L.; Umicevic Mirkov, M.; Boucher, G.; Anderson, C. A.; Andersen, V.; Cleynen, I.; Cortes, A.; Crins, F.; D'Amato, M.; Deffontaine, V.; Dmitrieva, J.; Docampo, E.; Elansary, M.; Farh, K. K.; Franke, A.; Gori, A. S.; Goyette, P.; Halfvarson, J.; Haritunians, T.; Knight, J.; Lawrance, I. C.; Lees, C. W.; Louis, E.; Mariman, R.; Meuwissen, T.; Mni, M.; Momozawa, Y.; Parkes, M.; Spain, S. L.; Theatre, E.; Trynka, G.; Satsangi, J.; van Sommeren, S.; Vermeire, S.; Xavier, R. J.; International Inflammatory Bowel Disease Genetics, C.; Weersma, R. K.; Duerr, R. H.; Mathew, C. G.; Rioux, J. D.; McGovern, D. P. B.; Cho, J. H.; Georges, M.; Daly, M. J.; Barrett, J. C., Fine-mapping inflammatory bowel disease loci to single-variant resolution. *Nature* **2017,** 547, (7662), 173-178.
9. Broquet, A. H.; Hirata, Y.; McAllister, C. S.; Kagnoff, M. F., RIG-I/MDA5/MAVS are required to signal a protective IFN response in rotavirus-infected intestinal epithelium. *Journal of immunology (Baltimore, Md. : 1950)* **2011,** 186, (3), 1618-26.
10. Jostins, L.; Ripke, S.; Weersma, R. K.; Duerr, R. H.; McGovern, D. P.; Hui, K. Y.; Lee, J. C.; Schumm, L. P.; Sharma, Y.; Anderson, C. A.; Essers, J.; Mitrovic, M.; Ning, K.; Cleynen, I.; Theatre, E.; Spain, S. L.; Raychaudhuri, S.; Goyette, P.; Wei, Z.; Abraham, C.; Achkar, J. P.; Ahmad, T.; Amininejad, L.; Ananthakrishnan, A. N.; Andersen, V.; Andrews, J. M.; Baidoo, L.; Balschun, T.; Bampton, P. A.; Bitton, A.; Boucher, G.; Brand, S.; Buning, C.; Cohain, A.; Cichon, S.; D'Amato, M.; De Jong, D.; Devaney, K. L.; Dubinsky, M.; Edwards, C.; Ellinghaus, D.; Ferguson, L. R.; Franchimont, D.; Fransen, K.; Gearry, R.; Georges, M.; Gieger, C.; Glas, J.; Haritunians, T.; Hart, A.; Hawkey, C.; Hedl, M.; Hu, X.; Karlsen, T. H.; Kupcinskas, L.; Kugathasan, S.; Latiano, A.; Laukens, D.; Lawrance, I. C.; Lees, C. W.; Louis, E.; Mahy, G.; Mansfield, J.; Morgan, A. R.; Mowat, C.; Newman, W.; Palmieri, O.; Ponsioen, C. Y.; Potocnik, U.; Prescott, N. J.; Regueiro, M.; Rotter, J. I.; Russell, R. K.; Sanderson, J. D.; Sans, M.; Satsangi, J.; Schreiber, S.; Simms, L. A.; Sventoraityte, J.; Targan, S. R.; Taylor, K. D.; Tremelling, M.; Verspaget, H. W.; De Vos, M.; Wijmenga, C.; Wilson, D. C.; Winkelmann, J.; Xavier, R. J.; Zeissig, S.; Zhang, B.; Zhang, C. K.; Zhao, H.; International, I. B. D. G. C.; Silverberg, M. S.; Annese, V.; Hakonarson, H.; Brant, S. R.; Radford-Smith, G.; Mathew, C. G.; Rioux, J. D.; Schadt, E. E.; Daly, M. J.; Franke, A.; Parkes, M.; Vermeire, S.; Barrett, J. C.; Cho, J. H., Host-microbe interactions have shaped the genetic architecture of inflammatory bowel disease. *Nature* **2012,** 491, (7422), 119-24.
11. Yang, E.; Shen, J., The roles and functions of Paneth cells in Crohn's disease: A critical review. *Cell Prolif* **2021,** 54, (1), e12958.
12. Negroni, A.; Colantoni, E.; Vitali, R.; Palone, F.; Pierdomenico, M.; Costanzo, M.; Cesi, V.; Cucchiara, S.; Stronati, L., NOD2 induces autophagy to control AIEC bacteria infectiveness in intestinal epithelial cells. *Inflamm Res* **2016,** 65, (10), 803-13.
13. Lee, C.; Choi, C.; Kang, H. S.; Shin, S. W.; Kim, S. Y.; Park, H. C.; Hong, S. N., NOD2 Supports Crypt Survival and Epithelial Regeneration after Radiation-Induced Injury. *Int J Mol Sci* **2019,** 20, (17).
14. Saxena, A.; Lopes, F.; Poon, K. K. H.; McKay, D. M., Absence of the NOD2 protein renders epithelia more susceptible to barrier dysfunction due to mitochondrial dysfunction. *Am J Physiol Gastrointest Liver Physiol* **2017,** 313, (1), G26-g38.
15. Wang, H.; Kim, J. J.; Denou, E.; Gallagher, A.; Thornton, D. J.; Shajib, M. S.; Xia, L.; Schertzer, J. D.; Grencis, R. K.; Philpott, D. J.; Khan, W. I., New Role of Nod Proteins in Regulation of Intestinal

Goblet Cell Response in the Context of Innate Host Defense in an Enteric Parasite Infection. *Infect Immun* **2016,** 84, (1), 275-85.
16. Siggers, R. H.; Hackam, D. J., The role of innate immune-stimulated epithelial apoptosis during gastrointestinal inflammatory diseases. *Cell Mol Life Sci* **2011,** 68, (22), 3623-34.
17. Fritz, T.; Niederreiter, L.; Adolph, T.; Blumberg, R. S.; Kaser, A., Crohn's disease: NOD2, autophagy and ER stress converge. *Gut* **2011,** 60, (11), 1580-8.
18. Ogura, Y.; Bonen, D. K.; Inohara, N.; Nicolae, D. L.; Chen, F. F.; Ramos, R.; Britton, H.; Moran, T.; Karaliuskas, R.; Duerr, R. H.; Achkar, J. P.; Brant, S. R.; Bayless, T. M.; Kirschner, B. S.; Hanauer, S. B.; Nunez, G.; Cho, J. H., A frameshift mutation in NOD2 associated with susceptibility to Crohn's disease. *Nature* **2001,** 411, (6837), 603-6.
19. Hugot, J. P.; Chamaillard, M.; Zouali, H.; Lesage, S.; Cezard, J. P.; Belaiche, J.; Almer, S.; Tysk, C.; O'Morain, C. A.; Gassull, M.; Binder, V.; Finkel, Y.; Cortot, A.; Modigliani, R.; Laurent-Puig, P.; Gower-Rousseau, C.; Macry, J.; Colombel, J. F.; Sahbatou, M.; Thomas, G., Association of NOD2 leucine-rich repeat variants with susceptibility to Crohn's disease. *Nature* **2001,** 411, (6837), 599-603.
20. Feng, Y.; Tsai, Y. H.; Xiao, W.; Ralls, M. W.; Stoeck, A.; Wilson, C. L.; Raines, E. W.; Teitelbaum, D. H.; Dempsey, P. J., Loss of ADAM17-Mediated Tumor Necrosis Factor Alpha Signaling in Intestinal Cells Attenuates Mucosal Atrophy in a Mouse Model of Parenteral Nutrition. *Mol Cell Biol* **2015,** 35, (21), 3604-21.
21. Fréour, T.; Jarry, A.; Bach-Ngohou, K.; Dejoie, T.; Bou-Hanna, C.; Denis, M. G.; Mosnier, J. F.; Laboisse, C. L.; Masson, D., TACE inhibition amplifies TNF-alpha-mediated colonic epithelial barrier disruption. *Int J Mol Med* **2009,** 23, (1), 41-8.
22. Hilliard, V. C.; Frey, M. R.; Dempsey, P. J.; Peek, R. M., Jr.; Polk, D. B., TNF-α converting enzyme-mediated ErbB4 transactivation by TNF promotes colonic epithelial cell survival. *Am J Physiol Gastrointest Liver Physiol* **2011,** 301, (2), G338-46.
23. Shimoda, M.; Horiuchi, K.; Sasaki, A.; Tsukamoto, T.; Okabayashi, K.; Hasegawa, H.; Kitagawa, Y.; Okada, Y., Epithelial Cell-Derived a Disintegrin and Metalloproteinase-17 Confers Resistance to Colonic Inflammation Through EGFR Activation. *EBioMedicine* **2016,** 5, 114-24.
24. Blaydon, D. C.; Biancheri, P.; Di, W. L.; Plagnol, V.; Cabral, R. M.; Brooke, M. A.; van Heel, D. A.; Ruschendorf, F.; Toynbee, M.; Walne, A.; O'Toole, E. A.; Martin, J. E.; Lindley, K.; Vulliamy, T.; Abrams, D. J.; MacDonald, T. T.; Harper, J. I.; Kelsell, D. P., Inflammatory skin and bowel disease linked to ADAM17 deletion. *N Engl J Med* **2011,** 365, (16), 1502-8.
25. Gettler, K.; Levantovsky, R.; Moscati, A.; Giri, M.; Wu, Y.; Hsu, N. Y.; Chuang, L. S.; Sazonovs, A.; Venkateswaran, S.; Korie, U.; Chasteau, C.; Duerr, R. H.; Silverberg, M. S.; Snapper, S. B.; Daly, M. J.; McGovern, D. P.; Brant, S. R.; Rioux, J. D.; Kugathasan, S.; Anderson, C. A.; Itan, Y.; Cho, J. H., Common and Rare Variant Prediction and Penetrance of IBD in a Large, Multi-ethnic, Health System-based Biobank Cohort. *Gastroenterology* **2021,** 160, (5), 1546-1557.
26. Nakatsu, F.; Baskin, J. M.; Chung, J.; Tanner, L. B.; Shui, G.; Lee, S. Y.; Pirruccello, M.; Hao, M.; Ingolia, N. T.; Wenk, M. R.; De Camilli, P., PtdIns4P synthesis by PI4KIIIα at the plasma membrane and its impact on plasma membrane identity. *J Cell Biol* **2012,** 199, (6), 1003-16.
27. Avitzur, Y.; Guo, C.; Mastropaolo, L. A.; Bahrami, E.; Chen, H.; Zhao, Z.; Elkadri, A.; Dhillon, S.; Murchie, R.; Fattouh, R.; Huynh, H.; Walker, J. L.; Wales, P. W.; Cutz, E.; Kakuta, Y.; Dudley, J.; Kammermeier, J.; Powrie, F.; Shah, N.; Walz, C.; Nathrath, M.; Kotlarz, D.; Puchaka, J.; Krieger, J. R.; Racek, T.; Kirchner, T.; Walters, T. D.; Brumell, J. H.; Griffiths, A. M.; Rezaei, N.; Rashtian, P.; Najafi, M.; Monajemzadeh, M.; Pelsue, S.; McGovern, D. P.; Uhlig, H. H.; Schadt, E.; Klein, C.; Snapper, S. B.; Muise, A. M., Mutations in tetratricopeptide repeat domain 7A result in a severe form of very early onset inflammatory bowel disease. *Gastroenterology* **2014,** 146, (4), 1028-39.
28. Jardine, S.; Dhingani, N.; Muise, A. M., TTC7A: Steward of Intestinal Health. *Cellular and molecular gastroenterology and hepatology* **2019,** 7, (3), 555-570.

29. Mohanan, V.; Nakata, T.; Desch, A. N.; Levesque, C.; Boroughs, A.; Guzman, G.; Cao, Z.; Creasey, E.; Yao, J.; Boucher, G.; Charron, G.; Bhan, A. K.; Schenone, M.; Carr, S. A.; Reinecker, H. C.; Daly, M. J.; Rioux, J. D.; Lassen, K. G.; Xavier, R. J., C1orf106 is a colitis risk gene that regulates stability of epithelial adherens junctions. *Science (New York, N.Y.)* **2018,** 359, (6380), 1161-1166.
30. Luong, P.; Hedl, M.; Yan, J.; Zuo, T.; Fu, T. M.; Jiang, X.; Thiagarajah, J. R.; Hansen, S. H.; Lesser, C. F.; Wu, H.; Abraham, C.; Lencer, W. I., INAVA-ARNO complexes bridge mucosal barrier function with inflammatory signaling. *eLife* **2018,** 7.
31. Rivas, M. A.; Beaudoin, M.; Gardet, A.; Stevens, C.; Sharma, Y.; Zhang, C. K.; Boucher, G.; Ripke, S.; Ellinghaus, D.; Burtt, N.; Fennell, T.; Kirby, A.; Latiano, A.; Goyette, P.; Green, T.; Halfvarson, J.; Haritunians, T.; Korn, J. M.; Kuruvilla, F.; Lagace, C.; Neale, B.; Lo, K. S.; Schumm, P.; Torkvist, L.; National Institute of, D.; Digestive Kidney Diseases Inflammatory Bowel Disease Genetics, C.; United Kingdom Inflammatory Bowel Disease Genetics, C.; International Inflammatory Bowel Disease Genetics, C.; Dubinsky, M. C.; Brant, S. R.; Silverberg, M. S.; Duerr, R. H.; Altshuler, D.; Gabriel, S.; Lettre, G.; Franke, A.; D'Amato, M.; McGovern, D. P.; Cho, J. H.; Rioux, J. D.; Xavier, R. J.; Daly, M. J., Deep resequencing of GWAS loci identifies independent rare variants associated with inflammatory bowel disease. *Nat Genet* **2011,** 43, (11), 1066-73.
32. Li, Y.; Jiang, Y.; Zhang, L.; Qian, W.; Hou, X.; Lin, R., Exogenous l-fucose protects the intestinal mucosal barrier depending on upregulation of FUT2-mediated fucosylation of intestinal epithelial cells. *FASEB journal : official publication of the Federation of American Societies for Experimental Biology* **2021,** 35, (7), e21699.
33. McGovern, D. P.; Jones, M. R.; Taylor, K. D.; Marciante, K.; Yan, X.; Dubinsky, M.; Ippoliti, A.; Vasiliauskas, E.; Berel, D.; Derkowski, C.; Dutridge, D.; Fleshner, P.; Shih, D. Q.; Melmed, G.; Mengesha, E.; King, L.; Pressman, S.; Haritunians, T.; Guo, X.; Targan, S. R.; Rotter, J. I., Fucosyltransferase 2 (FUT2) non-secretor status is associated with Crohn's disease. *Human molecular genetics* **2010,** 19, (17), 3468-76.
34. Wacklin, P.; Makivuokko, H.; Alakulppi, N.; Nikkila, J.; Tenkanen, H.; Rabina, J.; Partanen, J.; Aranko, K.; Matto, J., Secretor genotype (FUT2 gene) is strongly associated with the composition of Bifidobacteria in the human intestine. *PloS one* **2011,** 6, (5), e20113.
35. Ruhlemann, M. C.; Hermes, B. M.; Bang, C.; Doms, S.; Moitinho-Silva, L.; Thingholm, L. B.; Frost, F.; Degenhardt, F.; Wittig, M.; Kassens, J.; Weiss, F. U.; Peters, A.; Neuhaus, K.; Volker, U.; Volzke, H.; Homuth, G.; Weiss, S.; Grallert, H.; Laudes, M.; Lieb, W.; Haller, D.; Lerch, M. M.; Baines, J. F.; Franke, A., Genome-wide association study in 8,956 German individuals identifies influence of ABO histo-blood groups on gut microbiome. *Nat Genet* **2021,** 53, (2), 147-155.
36. Rausch, P.; Rehman, A.; Kunzel, S.; Hasler, R.; Ott, S. J.; Schreiber, S.; Rosenstiel, P.; Franke, A.; Baines, J. F., Colonic mucosa-associated microbiota is influenced by an interaction of Crohn disease and FUT2 (Secretor) genotype. *Proceedings of the National Academy of Sciences of the United States of America* **2011,** 108, (47), 19030-5.
37. Zhang, H.; Cui, Z.; Cheng, D.; Du, Y.; Guo, X.; Gao, R.; Chen, J.; Sun, W.; He, R.; Ma, X.; Peng, Q.; Martin, B. N.; Yan, W.; Rong, Y.; Wang, C., RNF186 regulates EFNB1 (ephrin B1)-EPHB2-induced autophagy in the colonic epithelial cells for the maintenance of intestinal homeostasis. *Autophagy* **2021,** 17, (10), 3030-3047.
38. Ji, Y.; Tu, X.; Hu, X.; Wang, Z.; Gao, S.; Zhang, Q.; Zhang, W.; Zhang, H.; Chen, W., The role and mechanism of action of RNF186 in colorectal cancer through negative regulation of NF-κB. *Cell Signal* **2020,** 75, 109764.
39. Fujimoto, K.; Kinoshita, M.; Tanaka, H.; Okuzaki, D.; Shimada, Y.; Kayama, H.; Okumura, R.; Furuta, Y.; Narazaki, M.; Tamura, A.; Hatakeyama, S.; Ikawa, M.; Tsuchiya, K.; Watanabe, M.; Kumanogoh, A.; Tsukita, S.; Takeda, K., Regulation of intestinal homeostasis by the ulcerative colitis-associated gene RNF186. *Mucosal Immunol* **2017,** 10, (2), 446-459.
40. Rivas, M. A.; Graham, D.; Sulem, P.; Stevens, C.; Desch, A. N.; Goyette, P.; Gudbjartsson, D.; Jonsdottir, I.; Thorsteinsdottir, U.; Degenhardt, F.; Mucha, S.; Kurki, M. I.; Li, D.; D'Amato, M.;

Annese, V.; Vermeire, S.; Weersma, R. K.; Halfvarson, J.; Paavola-Sakki, P.; Lappalainen, M.; Lek, M.; Cummings, B.; Tukiainen, T.; Haritunians, T.; Halme, L.; Koskinen, L. L.; Ananthakrishnan, A. N.; Luo, Y.; Heap, G. A.; Visschedijk, M. C.; Consortium, U. I. G.; Consortium, N. I. G.; MacArthur, D. G.; Neale, B. M.; Ahmad, T.; Anderson, C. A.; Brant, S. R.; Duerr, R. H.; Silverberg, M. S.; Cho, J. H.; Palotie, A.; Saavalainen, P.; Kontula, K.; Farkkila, M.; McGovern, D. P.; Franke, A.; Stefansson, K.; Rioux, J. D.; Xavier, R. J.; Daly, M. J.; Barrett, J.; de Lane, K.; Edwards, C.; Hart, A.; Hawkey, C.; Jostins, L.; Kennedy, N.; Lamb, C.; Lee, J.; Lees, C.; Mansfield, J.; Mathew, C.; Mowatt, C.; Newman, B.; Nimmo, E.; Parkes, M.; Pollard, M.; Prescott, N.; Randall, J.; Rice, D.; Satsangi, J.; Simmons, A.; Tremelling, M.; Uhlig, H.; Wilson, D.; Abraham, C.; Achkar, J. P.; Bitton, A.; Boucher, G.; Croitoru, K.; Fleshner, P.; Glas, J.; Kugathasan, S.; Limbergen, J. V.; Milgrom, R.; Proctor, D.; Regueiro, M.; Schumm, P. L.; Sharma, Y.; Stempak, J. M.; Targan, S. R.; Wang, M. H., A protein-truncating R179X variant in RNF186 confers protection against ulcerative colitis. *Nature communications* **2016,** 7, 12342.

41. Beaudoin, M.; Goyette, P.; Boucher, G.; Lo, K. S.; Rivas, M. A.; Stevens, C.; Alikashani, A.; Ladouceur, M.; Ellinghaus, D.; Törkvist, L.; Goel, G.; Lagacé, C.; Annese, V.; Bitton, A.; Begun, J.; Brant, S. R.; Bresso, F.; Cho, J. H.; Duerr, R. H.; Halfvarson, J.; McGovern, D. P.; Radford-Smith, G.; Schreiber, S.; Schumm, P. L.; Sharma, Y.; Silverberg, M. S.; Weersma, R. K.; D'Amato, M.; Vermeire, S.; Franke, A.; Lettre, G.; Xavier, R. J.; Daly, M. J.; Rioux, J. D., Deep resequencing of GWAS loci identifies rare variants in CARD9, IL23R and RNF186 that are associated with ulcerative colitis. *PLoS Genet* **2013,** 9, (9), e1003723.
42. Amarachintha, S.; Harmel-Laws, E.; Steinbrecher, K. A., Guanylate cyclase C reduces invasion of intestinal epithelial cells by bacterial pathogens. *Sci Rep* **2018,** 8, (1), 1521.
43. Garin-Laflam, M. P.; Steinbrecher, K. A.; Rudolph, J. A.; Mao, J.; Cohen, M. B., Activation of guanylate cyclase C signaling pathway protects intestinal epithelial cells from acute radiation-induced apoptosis. *Am J Physiol Gastrointest Liver Physiol* **2009,** 296, (4), G740-9.
44. Tronstad, R. R.; Kummen, M.; Holm, K.; von Volkmann, H. L.; Anmarkrud, J. A.; Hoivik, M. L.; Moum, B.; Gilja, O. H.; Hausken, T.; Baines, J.; Karlsen, T. H.; Fiskerstrand, T.; Hov, J. R., Guanylate Cyclase C Activation Shapes the Intestinal Microbiota in Patients with Familial Diarrhea and Increased Susceptibility for Crohn's Disease. *Inflammatory bowel diseases* **2017,** 23, (10), 1752-1761.
